# Endothelial Biomarkers and Cytokine Profiles: Signatures of Mortality in Severe COVID-19

**DOI:** 10.3390/ijms27031272

**Published:** 2026-01-27

**Authors:** Quintin A. van Staden, Muriel Meiring, Hermanus A. Hanekom, Vongani Nkuna, Lezelle Botes, Francis E. Smit

**Affiliations:** 1 Department of Haematology and Cell Biology, School of Pathology, Faculty of Health Sciences, University of the Free State, Bloemfontein 9300, South Africa; 2 Department of Haematology and Cell Biology, Universitas Academic Laboratories, National Health Laboratory Service, Bloemfontein 9300, South Africa; 3 Department of Cardiothoracic Surgery, School of Clinical Medicine, Faculty of Health Sciences, University of the Free State, Bloemfontein 9300, South Africa

**Keywords:** COVID-19, SARS-CoV-2, endothelium, biomarkers, cytokines, mortality

## Abstract

Severe acute respiratory syndrome coronavirus 2 (SARS-CoV-2) infection results in dysregulated inflammatory and coagulation pathways that drive immunothrombosis and contribute to adverse clinical outcomes. While individual cytokines and endothelial biomarkers have been associated with disease severity and mortality, the prognostic relevance of combined inflammatory and endothelial signatures remains incompletely characterised. To identify inflammatory cytokines and markers of endothelial activation associated with mortality in patients with severe COVID-19 requiring supplemental oxygen. This retrospective observational study included 73 consecutive adults admitted to a dedicated supplemental oxygen unit with severe COVID-19. Plasma concentrations of IL-1α, IL-1β, IL-6, IL-8, IL-10, TNF-α, von Willebrand factor (VWF) antigen and propeptide, ADAMTS13 antigen and activity, and ADAMTS13 autoantibodies were measured on admission using ELISA-based assays. Associations with mortality were assessed using non-parametric analyses, age-adjusted logistic regression, multivariable logistic regression, and receiver operating characteristic (ROC) curve analysis. Increasing age was independently associated with mortality. After adjustment for age, higher IL-1α concentrations were associated with increased odds of death, whereas a higher IL-6/IL-10 ratio was independently protective. In multivariable models, including non-ratio variables, ADAMTS13 autoantibody levels remained independently associated with mortality. In ratio-based multivariable analysis, both the ADAMTS13 activity/autoantibody ratio and the IL-6/IL-10 ratio were independently protective, while age was no longer significant. IL-10 and ADAMTS13 autoantibodies demonstrated moderate discriminative performance for mortality prediction (AUC ~0.70). A combined biomarker model incorporating IL-1α, IL-8, IL-10, and ADAMTS13 autoantibodies yielded very high predicted mortality probabilities. Our findings highlight IL-1α and ADAMTS13 autoantibodies as independent predictors of mortality in severe COVID-19, reflecting the interplay between inflammatory and endothelial pathways. Biomarker ratios capturing immune and endothelial balance—particularly the ADAMTS13 activity/autoantibody ratio—may enhance risk stratification and support integrated prognostic models.

## 1. Introduction

Severe acute respiratory syndrome coronavirus 2 (SARS-CoV-2) infection induces inflammatory and coagulation dysregulation that drives immunothrombosis, characterised by micro and macrovascular events [1]. This is supported by several meta-analyses and large case series that reported increased rates of venous and arterial events, associated with increased mortality in severe COVID-19, emphasising the significance of immunothrombosis on patient outcome [2]. Inflammatory cytokines are frequently elevated in COVID-19 and even more so in severe COVID-19 infection (defined here as requiring supplemental oxygen). Elevations in pro-inflammatory (IL-1, IL-6, IL-8, and TNF) markers and counter-regulatory cytokines (IL-10) typically reflect the immense immune activation and subsequent homeostatic attempt at anti-inflammatory control, likely induced by damage-associated molecular patterns [3,4].

Multiple studies have found correlations between circulating cytokines and hypoxia, as well as organ dysfunction in COVID-19 [4,5]. Efforts to develop predictive tools for COVID-19 severity and mortality have produced generally consistent patterns in clinical and biological markers, although definitive associations with adverse outcomes are still evolving in subgroups [6]. Variability in quantification and specific prognostic markers is largely attributable to differences in the number of interleukins assessed and the methods employed to quantify them [7]. Del Valle et al. demonstrated that increased admission values of IL-6, IL-8, and TNF-α are associated with increased mortality, with only IL-8 and TNF-a remaining significant with multivariate analysis [8], establishing these markers as prognostic indicators. Importantly, fewer large studies have investigated IL-1, known to play a central role in the biology of hypoxia [9], and the success of IL-1 blockade supports this mechanism’s role in COVID-19 [10]. A combination of cytokine markers has generally outperformed single markers in prognostic models. Unfortunately, a comparison of studies is hampered by heterogeneity of assays, sampling times and a lack of established cut-off values [11,12]. Prior studies have also emphasised the prognostic value of cytokine balances such as IL-6/IL-10 rather than absolute cytokine concentrations. These ratios have been proposed as superior prognostic indices, particularly in ICU cohorts [13].

Von Willebrand factor antigen, together with ADAMTS13, not only serve as biomarkers of endothelial activation but also provide insight into the dysregulated haemostatic balance in COVID-19. Direct evidence of endothelial activation is reflected by elevations in von Willebrand factor (VWF) antigen and, acutely, by VWF propeptide, with their imbalance contributing to a prothrombotic state. Multiple studies on COVID-19 have demonstrated that increased VWF markers are associated with higher oxygen requirements, ICU admission, and mortality, while an elevated VWF: ADAMTS13 ratio predicts platelet-rich thrombi and adverse clinical outcomes [14,15,16,17]. In addition, ADAMTS13 autoantibodies have been reported in approximately one-third of hospitalised COVID-19 patients, contributing to reduced ADAMTS13 activity and enhanced VWF-mediated immunothrombosis, which are associated with increased mortality. Consequently, several groups have advocated for the inclusion of VWF antigen, VWF propeptide, ADAMTS13 activity, and ADAMTS13 autoantibody testing in severe COVID-19 cohorts to aid risk stratification. Here again, there is growing advocacy for using ratios such as VWF antigen/ADAMTS13 activity and ADAMTS13 autoantibody levels/ADAMTS13 activity, which better reflect the coagulation balance in COVID-19 and are linked with disease severity and worse outcomes [15,16,17,18,19].

This study aimed to identify markers of endothelial activation (VWF antigen, ADAMTS13 activity, and ADAMTS13 autoantibodies), along with cytokine markers, that are associated with mortality in patients with severe COVID-19.

## 2. Results

Decoded abbreviations: COPD, Chronic Obstructive Pulmonary Disease (a chronic inflammatory lung disease causing airflow obstruction); HbA1c, Haemoglobin A1c (Glycated Haemoglobin, a blood test reflating average blood glucose over the past 2–3 months); BMI, Body Mass Index (a measure of body fat based on height and weight); BP, Blood Pressure (the pressure of circulating blood on the walls of blood vessels).

94.5% of patients had ADAMTS13 antibodies. There was a minor difference in VWF activity between the groups and a 17% difference in the median ADAMTS13 activity. The median VWF propeptide is notably increased in the ADAMTS13 antibody group (391 vs. 285). The number of patients without antibodies is too small (n = 4) to allow for more sophisticated analysis.

**Figure 1:**
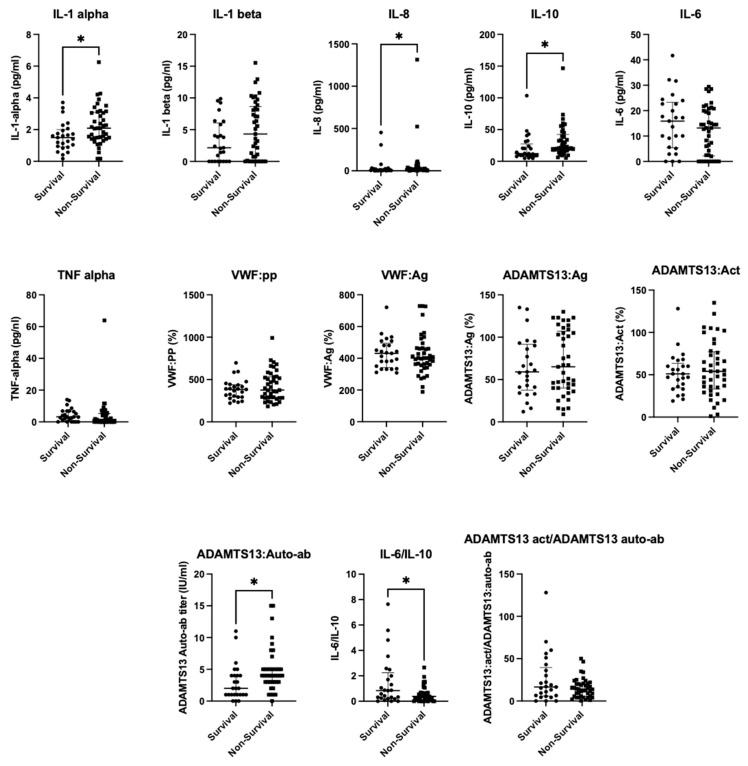
Box-and-whisker plots comparing survivors and non-survivors for each variable. * indicated statistical significance (*p* < 0.05).

Figure 1 shows that significant positive associations with death were observed for IL-1α (ρ = 0.30, weak–moderate), IL-8 (ρ = 0.24, weak), IL-10 (ρ = 0.34, weak–moderate), and ADAMTS13 autoantibody levels (ρ = 0.35, weak–moderate). In contrast, the IL-6/IL-10 ratio (ρ = −0.27, weak negative) and the ADAMTS13 activity/autoantibody ratio (ρ = −0.27, weak negative) were significantly associated with death. No significance was detected for all variables testing, including IL-1 alpha (pg/mL)/IL-10 (pg/mL), IL-1.beta (pg/mL)/IL-10 (pg/mL), VWF:Ag/ADAMTS13:Ag, and VWF:PP/VWF:Ag ratios. Consistent with the findings of the Spearman correlation, IL-1α, IL-8, IL-10, and ADAMTS13 autoantibody levels differed significantly between survivors and non-survivors. Significant differences were also observed for the ADAMTS13 activity/autoantibody and IL-6/IL-10 ratios.

Increasing age was associated with the outcome (OR 1.05, *p* = 0.007), as were higher IL-1α concentrations (OR 2.03, *p* = 0.022). In contrast, a higher IL-6/IL-10 ratio was inversely associated with the outcome (OR 0.49, *p* = 0.030).

Both age (OR 1.05, *p* = 0.043) and ADAMTS13 autoantibody levels (OR 1.39, *p* = 0.031) are independently associated with the outcome.

Among ratio variables, multivariate analysis demonstrated that the ADAMTS13 activity/autoantibody ratio (OR 0.94, *p* = 0.019) and the IL-6/IL-10 ratio (OR 0.40, *p* = 0.024) were independently associated with the outcome, whereas age was not significant in this model.

IL-10 and ADAMTS13 autoantibodies are the most promising markers for predicting mortality, although their performance is moderate (AUC ~0.70). IL-8 has the highest sensitivity (0.9), and IL-1α has the highest specificity (0.808). The ADAMTS13 activity/autoantibody ratio has a good sensitivity (0.95) and poor specificity (0.35).

Predicted mortality probabilities from single variable and multivariable logistic regression:

Very high predicted probabilities (>0.99) were observed when IL-1α (6.251525 pg/mL), IL-8 (1314.923 pg/mL), IL-10 (146.524 pg/mL), and ADAMTS13 (15) autoantibodies were combined and elevated at the above cut-offs.

## 3. Discussion

Our logistic regression analysis identified IL-1α and the IL-6/IL-10 ratio, as well as ADAMTS13 autoantibodies in the multivariate regression, as independent predictors of mortality in severe COVID-19, highlighting two complementary pathogenic pathways: cytokine-mediated inflammation and endothelial activation (Table 1, Table 2, Table 3, Table 4, Table 5, Table 6, Table 7 and Table 8).

The significance of IL-1α in our cohort is noteworthy, as few large studies have examined this cytokine directly or have not found an association with increased mortality [7,20]. Unlike IL-1β, which is inducible, IL-1α is constitutively expressed in endothelial cells and acts as a damage-associated molecular pattern (DAMP) under hypoxic stress [9,10,21]. This aligns with the profound endothelial activation in severe COVID-19, supported in our cohort by universal elevation of VWF propeptide. While IL-1β has been more consistently associated with poor outcomes [22], the early release of IL-1α may better capture acute endothelial injury. Our findings are consistent with a similarly sized study linking IL-1α to mortality risk [23]. In addition, when age was not controled, IL-8 and IL-10 were significantly associated with death on univariate analysis and demonstrate the important concept of inflammaging. Inflammaging, the chronic hyperinflammatory process that accompanies ageing, likely plays a role in this cohort. Interestingly, cytokines that are typically prominently increased, including interleukin-6 (IL-6) and tumour necrosis factor-α (TNF-α), were not elevated. The literature indicates that most COVID-19 cases occur in individuals aged 60 years and older, and, in conjunction with inflammaging, inflammatory responses become progressively less efficient, evolving into a state of immunosenescence, endothelial activation and coagulopathy. This likely contributes to the age-associated increase in mortality observed in this study cohort [24,25,26,27]. All co-morbidities mentioned in the descriptive analysis did not reach statistical significance and are not considered confounding (HIV was underpowered).

IL-8, a potent neutrophil chemoattractant, fits mechanistically with immunothrombosis [1,28], and a large study in New York found IL-8, along with IL-6 and TNF-alpha, as a strong independent predictor of survival [8]. IL-10, although traditionally regarded as anti-inflammatory, is increasingly recognised as a paradoxical proinflammatory marker in COVID-19 and may have a regulatory role in inflammation. This cytokine is known to be increased in COVID-19 patients in the ICU [29], with underlying mechanisms linked to immune dysfunction and impaired viral clearance [30]. Interestingly, although the cytokine combinations differ, the key cytokines identified in Ghaffarpour et al.’s study (IL-1Ra, IL-6, and IL-2) are similar to our findings in that they reflect a balance of pro-inflammatory and likely compensatory anti-inflammatory responses [22]. IL-Ra, established as a suppressor of IL-1, was used as the principle for the development of the IL-1 inhibitor, Anakinara, to treat COVID-19 [31].

IL-6-upregulation is well described in COVID-19 [32], but unlike many previous studies [8,22,33], IL-6 itself was not associated with mortality in our study. This may reflect different cohorts, the size of the tested cohort, sampling variability, or assay differences. Other possible causes include the influence of diurnal fluctuation of IL-6, which is lowest in the morning [34], inter-individual variability, and factors such as obesity and visceral fat deposition [35,36]. Ratios that include IL-6, such as IL-6/IL-10, are therefore seen as more robust indicators and are increasingly used as prognostic markers [37]. In our cohort, a higher IL-6/IL-10 ratio was independently protective against death. This contrasts with prior research, including the Dublin-Boston score, where an increasing IL-6/IL-10 ratio predicted deterioration [37]. Apart from pre-analytical factors such as cohort differences, timing of sampling, and assay variability, differences in the immune response to variant COVID-19 strains may also play a role. Additionally, either the purported pro-inflammatory properties of IL-10 were dominant or other cytokines, such as IL-1α, which are known to strongly induce IL-10, may have played a leading role, more so than IL-6, in driving IL-10 production. It is possible, although unlikely, that the IL-10 increase in our cohort was more sensitive to lower levels of IL-6, compared to international cohorts. This is partially in keeping with another South African study, which demonstrated an increase in IL-6 in COVID-19, associated with univariate analysis but not in multivariate analysis [38]. Our study and Mesecth et al.’s South African study support the findings and offer an explanation for the findings in a clinical trial of IL-6 inhibitor tocilizumab in an African population. In contrast to the success of the drug in international cohorts, there were higher rates of secondary infections and no significant improvement in the mortality rate of COVID-19 [39].

Endothelial activation is central to COVID-19 pathogenesis. As part of a complex of changes associated with endothelioathy, an increase in VWF antigen is associated with mortality in COVID-19 in a number of previous studies [1,40,41]. The challenge with this interpretation is that VWF is an acute-phase reactant protein, and it was unknown if this increase was simply a bystander inflammatory increase or due to activated endothelial damage with subsequent thrombosis. This was made clear after van den Berg et al. performed autopsies and conclusively proved that, in comparison to matched controls, VWF-rich thrombi were more common in COVID-19 and contributed to thrombosis in COVID-19 [42]. In keeping with Rostami et al.’s systemic review and meta-analysis of COVID-19 patients in the ICU, we observed a markedly elevated VWF antigen and propeptide levels in almost all patients with comparable estimates from meta-analyses of ICU cohorts (mean VWF:Ag ~388 IU/dL vs. median 401 IU/dL in our study; ADAMTS13 activity 58% vs. median 51%) [43,44]. In terms of ratios, previous studies have shown that reduced ADAMTS13 activity and elevated VWF:Ag/ADAMTS13 activity ratios predict adverse outcomes [15,16]. In contrast, our cohort did not show a significant difference in VWF levels between survivors and non-survivors, possibly due to sample size and the acute-phase reactivity of VWF.

A striking feature of our study is the high prevalence of ADAMTS13 autoantibodies (95%), which was independently associated with mortality. This extends the findings of Doevelaar et al., who reported ADAMTS13 autoantibodies in ~55% of critically ill patients [19], with similar medians of 7.5 ADAMTS13 autoantibody U/mL in critically ill patients, while ours is 3.8 U/mL [19]. However, in contrast to their findings, the ADAMTS13 antigen/VWF antigen ratio did not predict severity or mortality in our study. Notably, we calculated that the ADAMTS13 autoantibody/antigen ratio correlates with outcomes, with higher ADAMTS13 activity relative to autoantibody conferring protection against death. Lower ADAMTS13 activity, relative to higher autoantibody levels, promotes immunothrombosis, highlighting why preserved ADAMTS13 function, relative to autoantibodies, is protective. This ratio has not, to our knowledge, been previously published in COVID-19 cohorts. It may also provide a more accurate reflection of the endothelial–autoimmune interplay than VWF:Ag/ADAMTS13 ratios or single markers alone. Emerging reports of plasma exchange improving outcomes in severe COVID-19 support this mechanism and highlight the therapeutic implications [45]. These antibodies may also be non-neutralising, partially inhibitory or context-dependent, rather than fully blocking enzymatic activity. ADAMTS13 activity assays reflect systemic plasma activity but do not capture localised in vivo dysfunction [46]. In severe COVID-19, profound endothelial activation leads to the release of ultra-large von Willebrand factor multimers. Under these high-shear, VWF-rich conditions, even modest functional inhibition or antibody-mediated interference may be sufficient to tip the haemostatic balance towards microvascular thrombosis. ADAMTS13 autoantibodies may contribute to mortality through immune-mediated endothelial injury rather than direct enzymatic inhibition. Their presence likely reflects broader immune dysregulation, loss of tolerance, and autoantibody generation in severe COVID-19. The absolute ADAMTS13 activity did not differ between survivors and non-survivors; the ADAMTS13 activity/autoantibody ratio was independently protective, indicating that relative balance, rather than enzyme quantity alone, is clinically relevant [47]. It is important to notice that the inhibitory activity of ADAMTS13 autoantibodies was not measured due to the low antibody titers.

Our findings underscore the importance of multi-marker models. Single biomarkers such as IL-6 or VWF show modest predictive accuracy (AUC ~0.70 in our cohort), whereas combinations (IL-1α, IL-8, IL-10, and ADAMTS13 autoantibodies) yielded very high predicted probabilities (>0.99). This supports an integrative approach, combining cytokine and endothelial markers, to improve prognostic precision and potentially guide therapies.

Surprisingly few HIV-positive patients required admission to the severe COVID-19 ward, despite the prothrombotic and inflammatory nature of SARS-CoV-2 infection [47]. In South Africa, where HIV prevalence is high, worse outcomes had been anticipated; however, observational data suggested a paradoxical protective effect of HIV co-infection. Future studies are needed to clarify the mechanisms underlying this phenomenon.

## 4. Methodology

This was a retrospective observational study that included 73 patients who were admitted to the supplemental oxygen unit (House Idahlia) at Universitas Hospital with severe COVID-19, as part of an umbrella research project. Patients admitted to House Idahlia were recruited as part of the main study with the title, “Free State Supplemental Oxygen Project for COVID-19 Patients,” for which ethics approval (Ref no UFS-HSD2020/0507/3006) was granted in 2020. This main study allowed for the taking and storage of 73 blood samples for related analysis. These blood samples were tested for inflammatory markers by Professor Muriel Meiring from the Department of Hematology at the University of the Free State. Blood samples were taken on admission to the severe COVID-19 ward. Folders were reviewed, and all cases of mortality were deemed to have been directly caused by COVID-19, as per the World Health Organisation’s definition [48].

### 4.1. Study Population

Seventy-three consecutive patients who were admitted to the supplemental oxygen unit (House Idahlia) (HSREC number UFS-HSD2020/0507/3006) with severe COVID in 2020 were included in the study. Data was sourced from the Cardiothoracic Surgery department’s clinical database and patient files. Data on age, sex, ethnic group, comorbidities, HIV status, and clinical outcomes were collected for each patient. Seventy patients had complete clinical information and were included in the descriptive analysis of the cohort.

### 4.2. Inclusion Criteria

Patients in need of supplemental oxygen in the house Idahlia (high care unit) at Universitas Hospital (n = 73, consecutive sampling). Patients 18 years or older with confirmed COVID pneumonia (COVID PCR) requiring escalation of oxygen therapy after failing (SO2 < 90% or respiratory rate >30 breaths/min), none re-breather mask oxygen.

### 4.3. Exclusion Criteria

Patients under the age of 18 years.

### 4.4. Ethical Statement

Ethics approval was obtained from the University of the Free State’s (UFS) Health Science Research Ethics Committee. Reference number: UFS-HSD2020/0507/3006 as part of the umbrella project, the “Free State Supplemental Oxygen Project for COVID-19 Patients”.

### 4.5. Study Design

We performed a retrospective cohort study (n = 73) nested within the umbrella project, the “Free State Supplemental Oxygen Project for COVID-19 Patients”. Severe disease was defined as the requirement for supplemental oxygen, consistent with WHO and NIH clinical severity criteria [49]. Patients requiring oxygen were selected as they represent a clinically meaningful subgroup at higher risk of morbidity and mortality, making them particularly relevant for studying prognostic biomarkers. All participants were admitted to a dedicated COVID-19 unit where oxygen therapy was universally provided. Mortality outcomes were assessed through record review and were known for 67 patients. Only these patients were included in the non-parametric correlation analysis and multivariate analysis.

Samples were collected and tested as described in Section 4. All tests were performed in the Special Haemostasis Laboratory of the Department of Haematology and Cell Biology, UFS.

### 4.6. Laboratory Tests

#### 4.6.1. Interleukin Assays

Interleukin 1-alpha and beta, Interleukin-6, Interleukin-8, Interleukin-10, and Tumour Necrosis Factor Alpha were measured with ELISA kits from Elabscience (Houston, TX, USA) according to the instructions of the manufacturer. IL1-alpha, IL-1 beta, IL-8, and TNF-alpha had a detection range of 7.81–500 pg/mL, while IL-6 and IL-10 ‘s detection range was from 1.56–100 pg/mL. The sensitivity of IL1-alpha was 3.01 pg/mL, that of IL1-beta, IL-8, and TNF-alpha was 4.69 pg/mL, and the sensitivity of IL-6 and IL-10 was 0.94 pg/mL.

#### 4.6.2. VWF Antigen Levels

VWF:Ag levels were determined with an in-house ELISA assay. High-binding ELISA plates were coated with rabbit anti-human VWF antibodies (DAKO, Glostrup, Denmark and incubated overnight at 4 °C. The World Health Organisation (WHO) 6th International Standard plasma for VWF was used as a calibrator together with normal and pathological controls. The patient’s plasma samples and controls were double diluted in Phosphate Buffered Saline (PBS)/0.1% Tween-20 (PBST), and the study samples and controls were added to the coated plate, covered with parafilm, and incubated for 2 h at 36 °C. After incubation, the plates were washed 4 times with PBST. The detection polyclonal rabbit anti-human VWF conjugated to horseradish peroxidase (DAKO, Glostrup, Denmark) was added and incubated at room temperature for an hour, and the reaction was terminated with sulphuric acid. The absorbance was measured at 490–630 nm, and the VWF results were obtained from a reference curve. The normal VWF antigen range was 50–150%.

#### 4.6.3. VWF Propeptide Levels

An ELISA-based assay was used to determine the levels of VWF propeptide (VWFpp) in human plasma samples. The antibody pair (MW1639) from Cell Science^®^ (Newburyport, MA, USA) was used, and the results were read at 490–630 nm within 30 min using a Biotek Synergy HT and an ELISA reader (Biotek, El Segundo, CA, USA). The normal range of VWF:pp was 60–140%.

#### 4.6.4. ADAMTS13 Antigen and Activity

Technozyme^®^ ADAMTS13 activity/antigen fluorogenic ELISA kit (Technoclone^®^, Vienna, Austria) was utilised. This assay has a normal ADAMTS13 antigen and activity range of 50–150%, with a lower limit of detection of 2% for both ADAMTS13 antigen and activity results.

#### 4.6.5. ADAMTS13 Auto-Antibodies

The technozyme anti-ADAMTS13 autoantibody ELISA kit (Technoclone^®^, Vienna, Austria) was utilised to detect ADAMTS13-IgG autoantibodies. The kit is a standardised ELISA designed to detect ADAMTS13-IgG autoantibodies in human plasma. Results were interpreted as negative if ADAMTS13 autoantibody titer was <12 U/mL, borderline if 12–15 U/mL, and positive if >15 U/mL. Plasma samples from the cohort with suspected HIV-TTP with ADAMTS13 activity levels of less than 10% were tested for ADAMTS13 autoantibodies. The levels of autoantibodies in the HIV-cohort were also determined [48].

### 4.7. Statistical Analysis

Quantitative data were collected from the instrument for each test performed, and Microsoft Excel^®^ was used for descriptive statistical analysis. Continuous variables were summarised using medians and interquartile range as the data were not normally distributed. Ratios of relevant biomarkers, supported by the prior literature, were calculated to explore potential synergistic effects and interactions.

Data were imported from an Excel spreadsheet into R (version 4.0.5). Observations with missing values in the mortality outcome variable were excluded (n = 67). Univariate analysis between groups was performed using non-parametric methods: Spearman’s Rank-Order Correlation and the Wilcoxon Signed-Rank tests. These tests are robust to skewed data, appropriate for this sample size, and allow for comparison with related studies. However, these statistical tests do allow for the introduction of the influence of confounding variables such as age. To account for potential confounding by age, age-adjusted logistic regression models were used with mortality as the binary outcome. Each biomarker and biomarker ratio was first evaluated individually in separate age-adjusted logistic regression models. Subsequently, two multivariable logistic regression models including age were constructed: one incorporating all individual (non-ratio) biomarkers and another incorporating all biomarker ratios. This approach was used because including both the numerator and denominator of a ratio alongside the ratio itself in a single model would create collinearity and potentially bias regression [50].

In additional analyses, separate age-adjusted logistic regression models were fitted to evaluate the associations of obesity and diabetes with mortality. Results are reported as odds ratios with 95% confidence intervals, and statistical significance was defined as a two-sided *p*-value < 0.0.

Receiver operating characteristic curve analysis was used to assess the discriminative performance of identified statistically significant variables from the univariate analysis. The area under the curve was calculated as a measure of accuracy, with higher values reflecting better discrimination [51]. Optimal cut-off points were determined using Youden’s index, which identified the threshold values that optimise the balance of sensitivity and specificity [52].

Lastly, regression coefficients from the single variable multivariate logistic regression model were used to calculate a weighted composite risk score. To evaluate combined biomarker effects, a grid of plausible values spanning the observed ranges was generated, and predicted probabilities were calculated for each combination. The highest predicted probability was identified as the maximum value from this grid [53].

Analyses were performed using RStudio (R version 1.2.5042, © 2009–2020 RStudio, Inc. Posit, MA, USA) with the following packages: openxlsx (data import), dplyr (data manipulation), broom (model tidying), and pROC (ROC analysis).

## 5. Conclusions

This study demonstrates that IL-1α and ADAMTS13 autoantibodies are independent predictors of mortality in severe COVID-19, reinforcing the dual importance of inflammatory and endothelial pathways in disease progression. While IL-6 alone showed limited prognostic utility, the IL-6/IL-10 ratio provided more robust risk stratification and, unexpectedly, was protective in our cohort. The universal elevation of VWF and high prevalence of ADAMTS13 autoantibodies highlight endotheliopathy as a key driver of adverse outcomes. Importantly, our identification of the ADAMTS13 autoantibody/antigen ratio as a novel prognostic marker suggests a new avenue for risk stratification and potentially targeted therapy. These findings support an argument for biomarker models that integrate cytokine and endothelial markers.

### Limitations

This study has a number of limitations. The sample size was modest (n = 73), and outcomes were available for 67 patients, limiting statistical power. Second, this was a single-centre study, raising the possibility of population factors influencing biomarker profiles. Clinical covariates such as age, comorbidities, and HIV status were not incorporated into the analysis. These factors are known to influence outcomes in COVID-19 and may confound biomarker associations. The timing of sampling was not standardised (relative to when COVID-19 symptoms first appeared); cytokine levels such as IL-6 are subject to diurnal variation and dynamic changes during the disease course. VWF ristocetin cofactor activity was not measured, precluding assessment of functional VWF activity. Our study lacked a non-COVID-19 control group. The very small number of patients without ADAMTS13 autoantibodies (n = 4) restricted subgroup comparisons and interpretation of antibody-negative cases. Not knowing the causes of death, particularly if they were related to thrombosis, would have strengthened the study and allowed comparison of this outcome with tested markers of endothelial activation. HIV status was included, although very few patients were living with HIV, which precluded subgroup analysis.

## Figures and Tables

**Table 1:** Descriptive statistics of the cohort (n = 70).

Total Cohort with Clinical Information (n = 70)			Non Survivors (n = 43)			Survivors (n = 27)		
Variable	N *	% *	Variable	N **	% (of Non Survivors) **	Variable	N **	% (of Survivors) **
Age (median in years)	61		Age (median in years)	63		Age (median in years)	50	
Africans (A)	55	78.6	HbA1c (median)	6.5		HbA1c (median)	6.2	
Mixed (M)	11	15.7	D-dimer (median)	1.1		D-dimer (median)	0.78	
Caucasian (C)	4	5.7	Length of stay (median)	9		Length of stay (median)	7	
Male	28	40.0	Diabetes (known)	19	44.2	Diabetes (known)	11	40.7
Female	42	60.0	Africans (A)	36	83.7	Africans (A)	19	70.4
Diabetes (known)	30	42.9	Mixed (M)	4	9.3	Mixed (M)	7	25.9
HbA1c (median)	6.7		Caucasian (C)	3	7.0	Caucasian (C)	1	3.7
HbA1c ≥ 6.5%	33	56.9	Male	16	37.2	Male	12	44.4
Asthma/COPD	2	2.9	Female	27	62.8	Female	15	55.6
Never smoked	68	97.1	Obese	22	51.2	Obese	14	51.9
D-dimer (median)	1.3		HIV positive	7	63.6	HIV positive	3	60.0
Median Length of Stay (days)	7	7.0						
Obese	36	51.4						
HIV positive	10	66.7						

* Calculated as a percentage or median of the total of each recorded variable, ** Calculated as a percentage, with the denominator being the total number of patients with a known outcome (mortality) and the total number of patients with a variable.

**Table 2:** Descriptive analysis for all participants (n = 73).

Test Performed	Median	Mode	2.5th–97.5th Percentile	Number of Patients with a Result Above the Reference Interval (n)	Percentage of Results Above Reference Interval (%)	Normal Reference Interval
Il-1 alpha (pg/mL)	1.69	0.1733423	0.173–4.23	1	1	0–5 pg/mL
IL-1 beta (pg/mL)	2.88	0	0–12.64	29	40	0–5 pg/mL
IL-8 (pg/mL)	14.73	12.42596	0.33–477.79	10	14	<62 pg/mL
IL-10 (pg/mL)	19.62	9.555882	5.19 –84.06	73	100	<3.5 pg/mL
IL-6 (pg/mL)	13.64	0	0–31.84	29	40	5–15 pg/mL
TNF-alpha (pg/mL)	2.60	0	0–16.09	3	4	0–16 pg/mL
VWF:PP	379.38	292.8945	208.73–713.49	73	100	50–150%
VWF:Ag	401.00	383	253.45–727.7	72	98.6	50–150%
ADAMTS13:Ag (%)	60.91	120	13.95–131.05	42	57.53	>50%
ADAMTS13:Act (%)	51.00	20	8.2–124.1	39	53.42	>50%
ADAMTS13 auto-ab	3.80	1	0–13.61	1	1.37	<15 IU/mL (for TTP)

**Table 3:** Median VWF and ADAMTS13 activity.

	Median ADAMTS13 Activity (%)	Median VWF Activity (%)	VWF Propeptide
With ADAMTS13 antibodies (n = 69)	50	403	391
Without ADAMTS13 antibodies (n = 4)	67	398	285
Survivors (n = 26)	51	422	389
Non-survivors (n = 41)	54	401	375

**Table 4:** Spearman correlation with death as an outcome.

Variable	Spearman_rho	Spearman_p
Il-1 alpha (pg/mL)	0.30	0.015
IL-8 (pg/mL)	0.24	0.047
IL-10 (pg/mL)	0.34	0.005
ADAMTS13.auto-ab	0.35	0.004
IL-6 (pg/mL)/IL-10 (pg/mL)	−0.27	0.03
ADAMTS13:Act/ADAMTS13.auto-ab	−0.27	0.032

**Table 5:** Significant variables following logistic regression controlling for age **.

Variable	OR	CI_Lower	CI_Upper	*p*_Value
Age	1.05331722	1.01411394	1.094036	0.00726943
Il-1.alpha.(pg/mL)	2.02505011	1.10713522	3.70399916	0.02200016
IL-6 (pg/mL)/IL-10 (pg/mL)	0.49186428	0.25959905	0.931939	0.02954141

** All co-morbidities mentioned in the descriptive analysis did not reach statistical significance and are not considered confounding (HIV was underpowered).

**Table 6:** Significant variables following multivariate logistic regression for all non-ratio variables, including age.

Variable	OR	CI_Lower	CI_Upper	*p*
Age	1.05268536	1.00150857	1.10647727	0.04345762
ADAMTS13.auto.ab	1.39090971	1.03131607	1.87588448	0.03061423

**Table 7:** Significant variables following multivariate logistic regression for all ratio variables.

Variable	OR	CI_Lower	CI_Upper	*p*
Age	1.0382247	0.98801578	1.09098513	0.13800273
ADAMTS13 activity/ADAMTS13 autoantibody	0.94479855	0.90094174	0.99079026	0.0192047
IL-6 (pg/mL)/IL-10 (pg/mL)	0.40460651	0.18445367	0.88752058	0.02396231

**Table 8:** ROC analysis of biomarkers in predicting mortality.

Variable	AUC	Threshold	Sensitivity	Specificity
Il-1 alpha (pg/mL)	0.675	2.08	0.512	0.808
IL-8 (pg/mL)	0.644	5.99	0.902	0.423
IL-10 (pg/mL)	0.703	12.85	0.854	0.577
ADAMTS13 auto-ab	0.705	2.41	0.829	0.577
IL-6 (pg/mL)	0.592	15.66	0.683	0.538
IL 6 pg/mL/IL 10 pg/mL	0.657	0.76	0.80	0.538
ADAMTS13.Act/ADAMTS13 auto ab	0.6625	35.5	0.95	0.348

## Data Availability

Data can be made available on reasonable request.
